# Understanding key symptoms, side effects, and impacts of HR+/HER2- advanced breast cancer: qualitative study findings

**DOI:** 10.1186/s41687-019-0098-1

**Published:** 2019-02-07

**Authors:** Nina Galipeau, Brittany Klooster, Meaghan Krohe, Derek H. Tang, Dennis A. Revicki, David Cella

**Affiliations:** 1 Patient-Centered Outcomes, Adelphi Values, 290 Congress St., 7th Floor, Boston, MA 02210 USA; 2 Health Economics and Outcomes Research, Novartis Inc., One Health Plaza Bldg 345, East Hanover, NJ 07936 USA; 3 Outcomes Research, Evidera, 7101 Wisconsin Ave., Suite 1400, Bethesda, MD 20814 USA; 4 Northwestern University Feinberg School of Medicine, 633 N. Saint Clair St., 19th Floor, Chicago, IL 60611 USA

**Keywords:** Oncology, Advanced breast cancer, Signs and symptoms, Health-related quality of life, Impacts, Qualitative research

## Abstract

**Background:**

Patients with breast cancer experience a variety of disease symptoms and treatment-related side effects that can adversely affect functioning. The breast cancer experience may differ across disease stages and biomarker subtypes. This study identified relevant disease symptoms, treatment-related side effects, and physical functioning impacts in women with hormone receptor-positive (HR+)/human epidermal growth factor receptor 2-negative (HER2-) advanced breast cancer.

**Methods:**

One-on-one concept elicitation interviews were conducted with 15 patients with HR+/HER2- advanced breast cancer. Open-ended questions about patient experience with breast cancer and its treatment were drawn from a semi-structured interview guide. All interviews were audio-recorded and transcribed verbatim, and codes were used to document and organize patient descriptions of their experiences. Coded concepts were defined, supported by exemplary patient quotes, and analyzed for frequency of mention and saturation. Five interviews with experienced oncologists were conducted to supplement the concept elicitation interviews.

**Results:**

The average age of the patients was 66 years. Most (53%) self-identified as White/Caucasian; 40%, as Black/African American. Patients were reported to have metastases to bone (87%), liver (20%), lung (13%), skin (7%), and lymph nodes (7%). The most frequently reported disease-related concepts were fatigue and lump in breast (47% each) and pain (40%), while the most frequently reported treatment-related side effects were hair loss (67%), fatigue/tiredness (47%), and nausea (40%). Patients identified 10 concepts associated with both breast cancer and its treatment, including fatigue/tiredness, shortness of breath, weakness, and nausea. The most frequently reported impacts on physical function included working around home (73%), walking (73%), and cooking (53%). The most frequently reported symptoms and side effects from the expert perspective included fatigue, pain, and hot flashes (*n* = 5 each, 100%), while the ability to work (*n* = 5, 100%) and walk (*n* = 3, 60%) were reported impacts, aligning with those concepts reported by patients. The resulting concepts were organized into a patient-based conceptual model.

**Conclusions:**

Patients have varied experiences due to HR+/HER2- advanced breast cancer and its treatment, and commonly experience fatigue/tiredness, hair loss, general pain, lump in breast, and nausea, as well as impacts to physical functioning (specifically, activities of daily living and mobility).

**Electronic supplementary material:**

The online version of this article (10.1186/s41687-019-0098-1) contains supplementary material, which is available to authorized users.

## Background

For patients with hormone receptor positive (HR+)/human epidermal growth factor receptor-2 negative (HER2-) advanced (i.e., metastatic, locally advanced, or stage III or IV) breast cancer, standard treatments include endocrine monotherapy or targeted therapy with an anti-estrogen in patients with non-visceral or asymptomatic visceral tumors [1]. Disease process and associated treatments can give rise to numerous symptoms, typically differing based on site of metastasis and disease course, and adverse effects that negatively impact patients’ day-to-day lives [1]. Treatment goals for this patient population are to increase overall survival, delay disease progression, and improve or maintain quality of life [2].

Because treatment for HR+/HER2- advanced breast cancer is rarely curative, understanding the most relevant disease-related symptoms, treatment-related side effects, and impacts, particularly those on physical functioning, is essential for promoting and maintaining high levels of quality of care in this patient population. In order to evaluate treatment benefits from the patient perspective, it is necessary to understand the most important and relevant aspects of the HR+/HER2- advanced breast cancer experience.

In its 2009 Patient-Reported Outcome (PRO) Guidance to Industry, the United States Food and Drug Administration (FDA) acknowledged the value of the patient perspective in evaluating drug efficacy to support labelling claims [3]. In oncology specifically, the FDA has encouraged the evaluation of patient symptoms and functioning in addition to more traditional biomarkers such as tumor response and progression-free survival [4, 5]. This acknowledges that the impact of disease-related symptoms and adverse events resulting from drug therapies plays a role in patient functioning, particularly in regard to physical functioning [6]. From a measurement strategy perspective, prior work suggests that core contributors to patient health-related quality of life (HRQoL) include disease-related symptoms, treatment-related side effects, and physical functioning, and that each core contributor should be articulated and measured separately in order to best assess patient-reported effects of therapy [6]. Given the plethora of disease-related symptoms and potential treatment-related side effects in oncology, the inclusion of PRO measures assessing drug effects is an important and necessary step in incorporating meaningful patient perspectives and better understanding the outcomes of these clinical trials.

To direct and inform the research described in this paper, a targeted review of the scientific literature was first conducted to identify and describe the patient experience of advanced breast cancer from the perspective of the peer-reviewed literature. The literature review identified several qualitative studies that sought to provide findings on the relevant and important disease-related symptoms, treatment-related side effects, and impacts of breast cancer from the patient perspective. For example, two semi-structured interview studies and one study that involved essay writing all identified pain and lack of energy (fatigue) as key symptoms or side effects among women with metastatic breast cancer [7–9]. Other symptoms and side effects that women in these studies described included muscle weakness, nausea, vomiting, infections, hot flashes, and sexual dysfunction. Study participants also reported a number of breast-cancer related impacts, such as the ability to enjoy life, worry that the condition will get worse, concerns about appearance, and the ability to work and care for family. Further, another exploratory study of the psychosocial aspects of metastatic breast cancer endorsed similar impacts [10].

Although patient experiences with advanced breast cancer have been researched, there is a need to understand patient experiences across biomarkers, which can differ substantially due to various sites of metastases, disease history, and treatment options [7, 11]. Understanding experiences across biomarkers can inform selection of tools that reflect concerns specific to each population. The purpose of this article is to identify relevant disease symptoms, treatment-related side effects, and physical functioning impacts in women with HR+/HER2- advanced breast cancer specifically from the perspective of patients, as well as experienced oncologists, in order to inform patient-centered measurement strategies of drug efficacy and adverse events in this patient population.

## Methods

Qualitative concept elicitation interviews were conducted with patients with HR+/HER2- advanced (i.e., metastatic, locally advanced, or stage III or IV) breast cancer. Interviews with experienced oncologists were also conducted to supplement findings from the patient perspective.

### Qualitative patient interviews

Concept elicitation interviews were conducted to collect and document information about the important and relevant disease-related symptoms, treatment-related side effects, and physical functioning impacts from the perspective of patients with HR+/HER2- advanced breast cancer. Patients were also administered selected PRO measures to evaluate their understanding of the content; results of that activity are beyond the scope of the present paper and will be published elsewhere.

Approval to execute the study was received from Quorum Independent Review Board on 27 June 2016. All patients completed and signed statements of informed consent prior to their participation and, once enrolled, completed a Demographic Health and Information Form. A clinician-completed Subject Clinical Screener was used to collect clinical data about patients enrolled in the study.

#### Patient recruitment

Patients were recruited in person by clinicians at four different clinical sites in the United States (US) from June 2016 to December 2016. Clinicians reviewed patient charts and communicated with a third-party recruitment agency regarding patient eligibility. Patients were considered eligible for participation if they had clinical confirmation of HR+/HER2- advanced breast cancer not amenable to curative treatment by surgery or radiotherapy and were willing and able to participate in a 90-min interview. Additional clinical targets were identified (i.e., menopausal status, treatment experience, and metastatic site) to align the qualitative study population with clinical trials, as recommended by the FDA’s 2009 PRO guidance [3]. Recruitment targeted approximately 20 patient participants based on predictions about the number of participants required to achieve saturation (i.e., the point at which no new or relevant information is gained from additional interviews [12, 13]).

#### Conduct of patient interviews

One-on-one patient interviews were conducted in person or over the telephone. The interviews were approximately 90 min long, and the concept elicitation portion lasted approximately 45 min. Trained interviewers followed a semi-structured interview guide developed to solicit spontaneous responses through open-ended questions about the topics of disease-related symptoms, treatment-related side effects, and physical functioning impacts experienced by patients. The interview guide contained questions designed to allow patients to provide detailed descriptions of these topics, such as:

*“You mentioned [Symptom A]. Can you tell me more about this? Are [Symptom A] and [Symptom B] the same or different?”*

*“Can you describe any changes to your daily life, if any, that you have experienced due to [patient’s term for breast cancer]? If so, how? What do you think causes [the impact]?”*

Some follow-up questions drew from the literature review and interviews with experienced oncologists to ensure concerns specific to the population were captured:

*“Has your breast cancer spread to any other parts of your body? What symptoms do you have in [metastatic site]?”*

#### Data management, analysis, and presentation

Interviews were audio-recorded, transcribed, anonymized (i.e., identifying information was removed), and imported into ATLAS.ti, a computerized qualitative data analysis package (ATLAS.ti Scientific Software Development GmbH, Berlin, Germany) [14], to facilitate the storing, coding, and retrieval of qualitative data using Boolean operators [15]. Coding consisted of a researcher or “coder” identifying transcript text where a patient expressed a unique breast cancer-related symptom, treatment-related side effect, or physical functioning impact (i.e., a concept), highlighting that text, and then assigning the text to a code from the codebook, or developing a new code, that best characterized the concept. All three coders coded and then reviewed the first transcript together, and subsequently met to review each coded transcript to ensure consistency across the study sample. This process was ongoing until harmonization of the use of all codes within all transcripts was reached.

To evaluate saturation, disease-related symptom, treatment-related side-effect, and physical functioning impact concept emergence was documented across sets of successive interviews. The first 25% of interviews were compared with the next 25% of interviews to see what, if any, new concepts emerged, followed by comparing the third 25% against the first 50%, and the final 25% against the first 75% [16–18]. Saturation was considered reached when no new relevant data was expected to emerge from additional interviews (i.e., when ≥90% of sign, symptom, and treatment-related side-effect concepts had emerged in the first 75% of interviews).

To organize and catalog patients’ descriptions of their HR+/HER2- advanced breast cancer experience, concepts were analyzed qualitatively by examining specific words and phrases used by the patient regarding relevant characteristics of each concept (e.g., description of sensation, duration, location, severity, frequency, and attribution of the concept to disease and/or its treatment) and quantitatively by overall frequency of report (i.e., the number of patients who reported a concept as being relevant). The frequency with which a concept was reported by patients as most bothersome or occurring most frequently was also analyzed quantitatively. This approach allowed for the concepts that are important and relevant to the patients’ HR+/HER2- advanced breast cancer experience to emerge by observing and examining the patient voice. Data collected from the interviews were organized into concept description tables and were used to construct an HR+/HER2- advanced breast cancer conceptual model.

### Interviews with experienced oncologists

Concept elicitation interviews with five oncologists experienced in the area of advanced breast cancer were conducted to support the patient interview findings by defining and describing disease-related symptom concepts, treatment-related side-effect concepts, and impact concepts from the perspective of therapeutic area experts. Experienced oncologists were identified by study sponsor-employed clinicians. The interviews were conducted over the telephone by trained interviewers, followed a semi-structured interview guide, and were approximately 60 min in length. The interviews were audio-recorded, transcribed, anonymized, and analyzed using semi-quantitative and qualitative data analytic methods via ATLAS.ti Version 7.5 or later [14]. Concepts reported by at least three oncologists were considered to be the most frequently reported.

## Results

### Qualitative patient interviews

#### Study sample

A total of 15 patients participated in the interviews. Patients’ ages ranged from 45 to 87 years, with a mean age of 66 years (standard deviatio*n* = 12.4). Six patients (40%) self-identified as black or African American, eight (53%) self-identified as white/Caucasian, and one (7%) selected “Other” in response to race. The majority of patients reported completing some college or beyond (*n* = 13, 87%) and reported their overall health status as “good” or “fair” (*n* = 13, 87%) (Table 1).

**Table 1:** Patient interviews: Demographic and health information (self-reported)^a^

	Total (*N* = 15) n (%)^b^
Age (years)	
Range	45.3–87.6
Mean (standard deviation)	66.0 (12.4)
Gender	
Female	15 (100.0%)
Ethnicity	
No, not Spanish/Hispanic/Latino	14 (93.3%)
Yes, Mexican/Mexican American, Chicano	1 (6.7%)
Race	
Black or African American	6 (40.0%)
White/Caucasian	8 (53.3%)
Other	1 (6.7%)
Education	
High school diploma (or GED) or less	0 (0.0%)
Some college or certificate program	7 (46.7%)
College or university degree (two- or four-year)	5 (33.3%)
Graduate degree	1 (6.7%)
Other	2 (13.3%)
Living status	
Living with family or friends	13 (86.7%)
Living alone	2 (13.3%)
Annual household income	
Under $25,000	1 (6.7%)
$25,000 to $49,999	6 (40.0%)
$50,000 to $74,999	3 (20.0%)
$75,000 to $99,999	1 (6.7%)
$100,000 and over	1 (6.7%)
Prefer not to answer	3 (20.0%)
Work status^c^	
Working full-time	4 (26.7%)
Working part-time	2 (13.3%)
Homemaker	1 (6.7%)
Retired	7 (46.7%)
Unemployed	1 (6.7%)
On disability	1 (6.7%)
Health in general	
Excellent	0 (0.0%)
Very good	0 (0.0%)
Good	8 (53.3%)
Fair	5 (33.3%)
Poor	2 (13.3%)
Other health conditions^c^	
Heart disease	1 (6.7%)
High blood pressure	8 (53.3%)
High cholesterol	2 (13.3%)
Pain	6 (40.0%)
Muscle pain	4 (26.7%)
Neuropathic pain	2 (13.3%)
Other pain	1 (6.7%)
Diabetes	2 (13.3%)
*Type 2*	*2 (13.3%)*
Thyroid disease	1 (6.7%)
Depression/anxiety	6 (40.0%)
None	2 (13.3%)
Other	4 (26.7%)

^a^Information self-reported by subject on the Demographic and Health Information Form

^b^Unless other statistic indicated

^c^Responses are not mutually exclusive

Most patients were post-menopausal (*n* = 12, 80%) and had an Eastern Cooperative Oncology Group score of 0–1 (*n* = 12, 80%; Table 2). Six (40%) were refractory to treatment with non-steroidal aromatase inhibitors (NSAIs), tamoxifen, or fulvestrant. A few patients had received mammalian target of rapamycin (mTOR) inhibitor treatment (*n* = 3, 20%) and/or cyclin-dependent kinase (CDK 4/6) inhibitor treatment (*n* = 5, 33%). Finally, the majority of patients’ breast cancer had metastasized to the bone (*n* = 13, 87%).

**Table 2:** Patient interviews: Clinician-reported patient health information^a^

	Total (*N* = 15) n (%)^b^
Menopausal status	
Pre-menopausal	3 (20.0%)
*Not on gonadotropin-releasing hormone (GnRH) agonist treatment*	*3 (100.0%)* ^*c*^
Post-menopausal	12 (80.0%)
Mammalian target of rapamycin (mTOR) inhibitor treatment	
Yes	3 (20.0%)
No	12 (80.0%)
Recurrent or progressive disease refractory to non-steroidal aromatase inhibitor (NSAI), tamoxifen, or fulvestrant	
Yes	6 (40.0%)
No	9 (60.0%)
Eastern Cooperative Oncology Group score	
0	1 (6.7%)
1	11 (73.3%)
2	3 (20.0%)
Cyclin-dependent kinase (CDK4/6) inhibitor treatment	
Yes	5 (33.3%)
No	10 (66.7%)
Metastatic site^d^	
Bone	13 (86.7%)
Lung	2 (13.3%)
Liver	3 (20.0%)
Lymph node	1 (6.7%)
Skin	1 (6.7%)

^a^Information reported by clinician on the Subject Clinical Screener

^b^Unless other statistic indicated

^c^This characteristic was only reported by the clinician for subjects who were pre-menopausal at diagnosis. The clinician for one subject reported menopausal status at the time of the study (post-menopausal); however, it was determined that treatment caused the subject to begin menopause. Thus, the subject is characterized as pre-menopausal. Data for this subject regarding treatment with a GnRH agonist were not provided

^d^Responses are not mutually exclusive

#### Disease-related symptom and treatment-related side-effect concepts

A total of 50 sign, symptom, and treatment-related side-effect concepts were elicited spontaneously from patients during the qualitative interviews. Patients identified 14 unique concepts that they attributed to be disease-related signs or symptoms; fatigue and lump in breast (*n* = 7 each, 47%) and pain (*n* = 6, 40%) were the three most frequently reported. Patients also identified 43 concepts that they attributed to be treatment-related side effects. The three most frequently reported treatment-related side effects were hair loss (*n* = 10, 67%), fatigue/tiredness (*n* = 7, 47%), and nausea (*n* = 6, 40%). Fatigue/tiredness, general pain, nausea, shortness of breath, weakness, diarrhea, lymphedema, weight loss, neck swelling, and cough were identified as both disease- and treatment-related experiences. Due to some overlap in patients’ attribution of disease- and treatment-related concepts, analyses were performed to identify the most frequently reported concepts overall, which included fatigue/tiredness (*n* = 12, 80%), hair loss (*n* = 10, 67%), and general pain and lump in breast (*n* = 7 each, 47%). The overall most frequently reported concepts are reported with exemplary patient quotes in Table 3.

**Table 3:** Patient-reported HR+/HER2- advanced breast cancer disease-and treatment-related concepts reported by ≥33.3% of patients (frequencies and descriptions)

Concept	Concept description^a^	*Frequency of patient report*^b^ *N* = 15 n (%)	Most bothersome to patient^c^ *N* = 15 n (%)	Most frequent to patient^d^ *N* = 15 n (%)	Attributed to disease or treatment by patient^e^ *N* = 15 n (%)
Fatigue/tiredness	“Um, now there are days when you’re really tired or you wake up and you feel weak. But it’s not every day. It’s just some days that you’re, you’re tired and you’re weak.” (01–01-F-79)	12 (80.0%)	2 (13.3%)	1 (6.7%)	Disease-related: 7 (46.7%) Treatment-related: 7 (46.7%) Unable to attribute: 0 (0.0%)
Hair loss	“I lost all of my hair. See, it’s coming back up there. I’m still losing it. … And my hair came back totally different. It used to be red. … You know, and now it’s like dark. *Q: Uh-huh.* A: You know, and kind of curly, and it was never curly, and so it came back totally different.” (01–07-F-45)	10 (66.7%)	1 (6.7%)	1 (6.7%)	Disease-related: 0 (0.0%) Treatment-related: 10 (66.7%) Unable to attribute: 0 (0.0%)
General pain	“I say bad, you know, just – well the only thing that really, really hurt me a lot was my legs. My legs were just – it, it felt to where I could barely walk sometimes. … Towards the end of the treatment it would get a little better. But even after the treatment stopped, um, the pain in my legs were so bad I had to end up going back to the doctor to see maybe it was something else.” (01–02-F-70)	7 (46.7%)	2 (13.3%)	3 (20.0%)	Disease-related: 4 (26.7%) Treatment-related: 4 (26.7%) Unable to attribute: 1 (6.7%)
Lump in breast	“I found the lump myself and, um, putting on my deodorant and I saw an inversion in my breast and I knew that was a sign of breast cancer. … the first one I felt was like hard as a rock. … I could see them. They were out. They weren’t in, you know, where you could just feel it. They were out. They were popping out.” (01–10-F-68)	7 (46.7%)	0 (0.0%)	0 (0.0%)	Disease-related: 7 (46.7%) Treatment-related: 0 (0.0%) Unable to attribute: 0 (0.0%)
Nausea	“Um, with the chemo I was nauseated, um, a lot. I didn’t want to eat because of the, um, smells of the food. And it just was hard to keep down. … It just feels like I, I have to, um, throw up. But it’s just, um, put like this – I don’t know. Like this – you know how when you, um, you smell some food that don’t smell good to your taste and it be like, ugh, and sometimes it makes your stomach quiver. That’s how the nausea makes my stomach feel. And it feels like I want to throw up, but it’s like it’s just stuck right in my throat, in my chest area.” (01–07-F-45)	6 (40.0%)	0 (0.0%)	2 (13.3%)	Disease-related: 1 (6.7%) Treatment-related: 6 (40.0%) Unable to attribute: 0 (0.0%)
Shortness of breath	“You know, because I did have shortness of breath, but that was with the drip chemo. … Because I would get to where I couldn’t talk, you know, and it would like it would be hard to breathe, and I’d have to stop, you know, and get – you’re making me experience all this stuff again. … Ah, but you know, it was like I couldn’t breathe, and then I would have to stop and then, you know, catch my breath to be able to talk again, and it would last about a week, you know.” (01–05-F-62)	6 (40.0%)	3 (20.0%)	2 (13.3%)	Disease-related: 3 (20.0%) Treatment-related: 2 (13.3%) Unable to attribute: 0 (0.0%)
Weakness	“But then, you know, you get up and then you have to sit back down. … don’t know what happened because I had done chemo like two months and then I just felt weak all over. I don’t know what happened, but, uh, they had put me in a wheelchair because I was never in a wheelchair before. I couldn’t sit up. I remember leaning over in the chair. I, I was dying. I was out. I couldn’t sit up. I couldn’t use my cell phone to call my family. I was just weak and tired.” (03–01-F-70)	5 (33.3%)	1 (6.7%)	1 (6.7%)	Disease-related: 1 (6.7%) Treatment-related: 4 (26.7%) Unable to attribute: 1 (6.7%)

^a^Concept descriptions are based on aggregated quotes from the total sample

^b^Frequency is presented as the total count for each concept reported at least once by at least one-third of patients; all signs, symptoms, and side effects were spontaneously reported by the patient without prior mention by the interviewer. The following concepts were reported by fewer than one-third of patients: diarrhea, lymphedema, neuropathy, skin burn, altered taste, constipation, feeling unwell, injection site reaction, joint pain, loss of appetite, vomiting, bone pain, dizziness, headache, hot flashes, indent in breast, memory loss, mouth sores, nail issues, neck swelling, stiffness, stomach pain, acid reflux, allergic reaction, bleeding, bloating, breast size decrease, chemotherapy brain, cough, flu-like symptoms, lung fluid build-up, flushing, gout, itching, lack of balance, lymph node inflammation, runny nose, skin peeling, sore throat, vaginal bleeding, weight gain (Additional file: 2 Table S1)

^c^Frequency is presented as the total count for each concept reported as most bothersome by the patients

^d^Frequency is presented as the total count for each concept reported as most frequent by the patients

^e^Frequency is presented as the total count for each concept reported as disease-related, treatment-related, and/or unsure attribution; frequency counts for concept attribution are not mutually exclusive

Eleven of the 50 sign, symptom, and treatment-related side effect concepts were reported by patients as the “most frequent” (i.e., occurring often) characteristic of their disease, with general pain reported by the greatest number of patients as occurring frequently (*n* = 3, 20%). The disease-related symptoms or treatment-related side effects that patients reported to be “most bothersome” were varied; shortness of breath (*n* = 3, 20%), followed by fatigue/tiredness, general pain, and vomiting (*n* = 2 each, 13%) were mentioned as “most bothersome” by more than one patient each. All of the “most frequent” concepts were also reported by patients as the most bothersome, with the exception of vomiting (Note: The “vomiting” concept is not included in Table 3, as it was reported by fewer than 20% of patients).

#### Impact concepts

The majority of the 36 total impact concepts reported by patients were related to the impact of HR+/HER2- advanced breast cancer on patients’ ability to function physically (*n* = 26, 72%). Most frequently, patients reported that their condition affected concepts within the activities of daily living domain (including their ability to do housework [*n* = 11, 73%] and ability to cook [*n* = 8, 53%]) and the general physical functioning domain (ability to walk [*n* = 11, 73%]) (Table 4). Patients spontaneously reported on other types of impacts aside from physical functioning, which were noted during analysis. For instance, more than a quarter of subjects described the impact their condition had on the domains of leisure activities (e.g., bowling) (*n* = 10, 67%), relationships (e.g., ability to maintain relationships) (*n* = 7, 47%), work (e.g., having to transition from full-time to part-time work) (*n* = 4, 27%), and sleep (e.g., difficulty sleeping due to pain or discomfort) (*n* = 4, 27%) (Additional file: 1 Table S3).

**Table 4:** Patient-reported HR+/HER2- advanced breast cancer physical function impact concept frequencies and descriptions reported by > 33.3% of patients

Concept	Concept description^a^	Frequency of patient reports *N* = 15 n (%)^b^	Associated sign, symptom, or treatment-related side effect N = 15 n(%)^c^
Instrumental activities of daily living (ADLs)			
Ability to do housework	“I don’t want to do nothing. Eat, cook, clean, nothing. I’m just that tired and I’ll go right to sleep. And my significant other, he be like, You can’t be that tired. Because he’s accustomed to us being active. … Or like if I’m doing stuff around the house like I might – say today I got up and I was off. I might say, oh I’m going to mop, mop up the house today. I haven’t mopped in a couple of days. And I might start mopping, but I might just done one little area and I’m feeling like exhausted. Now I don’t want to mop anymore. So sometimes I have to sit down and take a rest and then finish up or sometimes I just get so aggravated with just being tired and can’t finish and just go ahead and finish up.” (01–02-F-70)	11 (73.3%)	Fatigue/tiredness 6 (40.0%)
			Lymphedema 2 (13.3%)
			Shortness of breath 2 (13.3%)
			General pain 1 (6.7%)
			Joint pain 1 (6.7%)
			Memory loss 1 (6.7%)
Ability to cook	“When I’m cooking with the hands, I can’t turn, um, can openers and stuff like that, um-hum, manual can openers. That it causes me to cook less. Uh, I had a catering business at one time. So I know that I’m a very good cook but now, in the kitchen, that’s tiresome.” (01–03-F-62)	8 (53.3%)	Fatigue/tiredness 5 (33.3%)
			Neuropathy 1 (6.7%)
			Weakness 1 (6.7%)
Ability to shop	“And my rest of my family was helping with the other things, you know, going to the store and so forth. But like I said, it was really, really a weak, weak time in my life.” (01–02-F-70)	6 (40.0%)	Fatigue/tiredness 3 (20.0%)
			Shortness of breath 2 (13.3%)
			Weakness 1 (6.7%)
General physical functioning			
Ability to walk	“I mean there are days I might have done – I might have walked a long distance. I don’t do that anymore. I walk a shorter distance. But as far as like my habits like gardening and planting flowers and going to the shopping center, it doesn’t – it hasn’t affected me. … After chemo and radiation, you become tireder quicker. So you don’t want to walk long distances unless you’re with someone. Even if you’re with someone you don’t want to walk long distances because you become tireder quicker.” (01–04-F-67)	11 (73.3%)	Fatigue/tiredness 5 (33.3%)
			Shortness of breath 4 (26.7%)
			Neuropathy 3 (20.0%)
			Weakness 2 (13.3%)
			Gout 1 (6.7%)
			Feeling unwell 1 (6.7%)

^a^Concept descriptions are based on aggregated quotes from the total sample

^b^Frequency is presented as the total count for each concept reported at least once by at least one third of patients; all impacts were spontaneously reported by the patient without prior mention by the interviewer. The following physical functioning impact concepts were reported by less than one-third of patients: toileting, bathing, ability to drive, ability to lift, ability to climb stairs, ability to exercise, ability to lie down, ability to sit, ability to stand, fine motor skills, ability to bend, ability to get in and out of car, ability to move at normal pace, ability to stand from seated position, dancing, gardening, bowling, crocheting/knitting, ability to care for pets, ability to play pool, lack of interest in hobbies, and ability to sleep (Additional file: 3 Table S2)

^c^Frequency is presented as the total count for each concept associated with a disease-related sign or symptom and/or treatment-related side effect

**Fig. 1:**
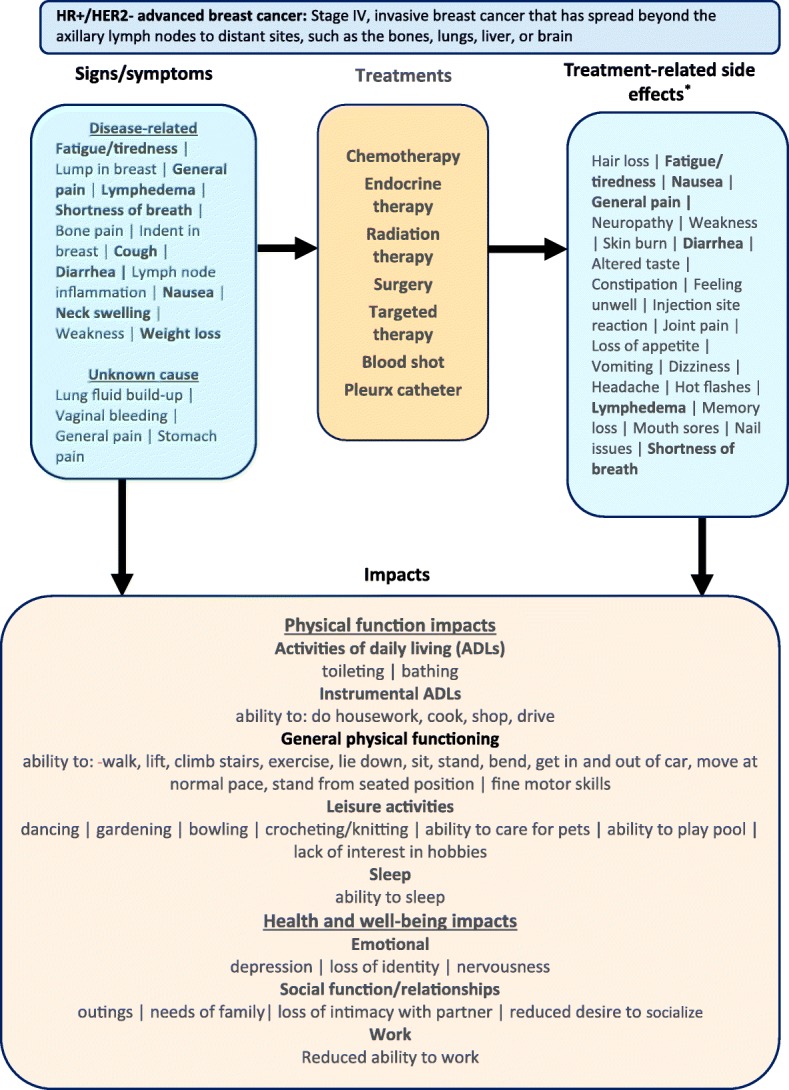
Patient-centric conceptual model of HR+/HER2- advanced breast cancer. Concepts that are shown in bold were reported by subjects patients to be related to both the disease and its treatment. Disease-related, treatment-related, and unknown cause concepts are ordered by frequency with which subjects patients reported concept attribution.*The following treatment-related side effects were reported by one subject patient each: acid reflux, allergic reaction, bleeding, bloating, breast size decrease, chemotherapy brain, cough, flu-like symptoms, flushing, gout, itching, lack of balance, neck swelling, runny nose, skin peeling, sore throat, stiffness, stomach pain, weight gain, weight loss

The 50 sign, symptom, and side effect concepts and 36 impact concepts reported by subjects during the concept elicitation portion of the interviews were organized into a patient-based conceptual model (Fig. 1).

#### Saturation

Of the 50 sign, symptom, and treatment-related side-effect concepts that were elicited spontaneously from subjects during the qualitative interviews, 48 (96%) were elicited in the first 75% of interviews. A total of 26 physical function impacts were elicited from subjects, 23 (88%) of which were elicited in the first 75% of interviews. Based on these saturation results, it was determined that an adequate number of interviews had been conducted.

### Interviews with experienced oncologists

Five experienced oncologists specializing in breast cancer participated in the expert interviews. Oncologists described 26 disease-related signs and symptoms relevant to patients with HR+/HER2- advanced breast cancer; the most frequently reported were fatigue and pain (*n* = 5 each, 100%), followed by neurologic symptoms (confusion, dizziness, dysarthria, headaches, seizures, trouble walking, vision disturbances, etc.) and shortness of breath (*n* = 4 each, 80%). In relation to treatment-related side effects, a total of 37 concepts were identified; the most frequently reported were fatigue (*n* = 5, 100%), hot flashes (*n* = 5, 100%), arthralgia (*n* = 4, 80%), hair loss (*n* = 4, 80%), mouth sores (*n* = 4, 80%), neuropathy (*n* = 4, 80%), and vaginal dryness (*n* = 4, 80%). Oncologists reported that both disease-related symptoms and treatment-related side effects can lead to a variety of impacts on patients, most notably physical functioning, and specifically the ability to work (*n* = 5, 100%), ability to walk (*n* = 3, 60%), and decreased motor function (*n* = 3, 60%). The impact on social functioning/relationships was also frequently discussed, such as the ability to engage with family and friends (*n* = 5, 100%) (Table 5).

**Table 5:** Oncologist-reported HR+/HER2- advanced breast cancer concepts (*N* = 5)

Concept	Disease symptom oncologist report n (%)	Treatment side effect oncologist report n (%)
Fatigue	5 (100.0%)	5 (100.0%)
Pain	5 (100.0%)	2 (40.0%)
Shortness of breath	4 (80.0%)	1 (20.0%)
Nausea/vomiting	4 (80.0%)	1 (20.0%)
Neurologic symptoms	4 (80.0%)	–
Weakness	3 (60.0%)	–
Loss of appetite	2 (40.0%)	3 (60.0%)
Hot flashes	–	5 (100.0%)
Arthralgia *Also referred to as joint pain*	–	4 (80.0%)
Mouth sores	–	4 (80.0%)
Neuropathy	–	4 (80.0%)
Vaginal dryness	–	4 (80.0%)
Diarrhea	–	3 (60.0%)

### Overall results

A concept tracking matrix was developed to evaluate the extent to which patients’ most frequently reported HR+/HER2- advanced breast cancer-related concepts were also endorsed by experienced oncologists (Table 6). In summary, all of the sign, symptom, and side effect concepts frequently reported by patients were also reported by the oncologists: fatigue/tiredness, hair loss, general pain, lump in breast, nausea, shortness of breath, and weakness. Additionally, fatigue/tiredness, general pain, nausea, and shortness of breath were identified as both disease- and treatment-related by patients and oncologists. Hair loss was reported by both sources as a treatment-related side effect only. Oncologists defined “weakness” as specifically a disease-related symptom, whereas the patients attributed weakness to both the disease and its treatment (Table 7). Of the three physical functioning impacts most frequently reported by patients, two were supported by the oncologists: ability to walk and ability to do housework. The third impact, patients’ ability to cook, was reported by approximately half of the patients (53.3%), but was not reported by the oncologists.

**Table 6:** Concept tracking matrix: Concepts reported by patients and experienced oncologists

Concepts^a^	Reported by patients *N* = 15 n (%)	Reported by oncologists
Signs, symptoms, and side effects		
Fatigue/tiredness	12 (80.0%)	✓
Hair loss	10 (66.7%)	✓
General pain	7 (46.7%)	✓
Lump in breast	7 (46.7%)	✓
Nausea	6 (40.0%)	✓
Shortness of breath	6 (40.0%)	✓
Weakness	5 (33.3%)	✓
Physical functioning impacts		
Ability to walk	11 (73.3%)	✓
Ability to do housework	11 (73.3%)	✓
Ability to cook	8 (53.3%)	
Ability to shop	6 (40.0%)	✓

^a^All concepts were reported by at least 33.3% of patients during the concept elicitation portion of interviews

**Table 7:** Attribution of concepts reported by the patients compared to oncologist reports

Concept^a^	Patients		Oncologists	
	Disease-related symptom	Treatment-related side effect	Disease-related symptom	Treatment-related side effect
Fatigue/tiredness	✓	✓	✓	✓
Hair loss		✓		✓
General pain	✓	✓	✓	✓
Lump in breast	✓		✓	
Nausea	✓	✓	✓	✓
Shortness of breath	✓	✓	✓	✓
Weakness	✓	✓	✓	

^a^All concepts were reported by at least 33.3% of patients during the concept elicitation portion of interviews

## Discussion

The intent of the present research was to identify the important and relevant disease-related symptoms, treatment-related side effects, and physical functioning impacts from the perspective of patients with HR+/HER2- advanced breast cancer, supported by the perspective of experienced oncologists. The present study suggests, similar to other research [7–9], that pain and fatigue were the most relevant symptoms. Further, these symptoms affect important areas of functioning [7]. The importance of fatigue specifically is consistent with findings in general advanced cancer populations. In a study by Butt et al. (2008) of symptoms in advanced cancer patients who had received chemotherapy for advanced bladder, brain, breast, colorectal, head and neck, hepatobiliary/pancreatic, kidney, lung, ovarian, or prostate cancer or lymphoma (*N* = 534), patients reported fatigue as either the first or second most important symptom or concern to monitor [19]. Garcia et al. also found fatigue to be the most important symptom experienced by advanced breast cancer patients [7]. Similarly, in Siefert’s 2010 study of outpatient chemotherapy patients (breast, colorectal, lung, and lymphoma; *N* = 70), fatigue was the most commonly reported symptom and most important to patients [20].

The present study also found that patients attributed many concepts to both the condition and its treatment, and in some cases, patients were unable to attribute a particular concept specifically to their disease or its treatment. The challenges of distinguishing between disease- and treatment-related symptoms were not unique to the current study or to breast cancer. Indeed, prior research by Cleeland et al. (2010) has noted that cancer-related disease and treatment symptoms do not occur in isolation, but instead occur simultaneously, and that this should be taken into consideration when trying to measure the patient experience [21]. Rosenberg and colleagues (2012) conducted a study that aimed to describe symptom burden and symptom attribution patterns in a large sample of breast cancer survivors taking endocrine therapy [22]. They found that more than half of women (53%) misattributed at least one symptom to their endocrine therapy.

The results of the current research were combined into a concept tracking matrix (Table 6), which demonstrates the extent to which patients’ most frequently reported disease-experience concepts overlapped with those from the experienced oncologists. The value of this table is that these results may help to guide future PRO instrument development for this specific patient population. While FDA PRO guidance places primacy on the patient perspective [3], understanding and evaluating the commonalities in report across the two data sources provides additional insight into which concepts may be the most important and relevant for future measurement.

Limitations of this study include several challenges in generalizing findings to the wider advanced breast cancer population. Although saturation was determined to have been achieved for symptoms/side effects, suggesting that these concepts are the key features of the HR+/HER2- advanced breast cancer experience, there is a possibility that because of the specific breast cancer subtype and treatments received, these results may not be generalizable to the wider advanced breast cancer population. Treatment-related concepts may vary based on treatment regimen, dose, sequence, and schedule [6]. Furthermore, it is not known the extent to which molecular subtype or solid tumor type affect the experience of disease-related symptoms. For example, it is not clearly understood whether HR-/HER2+ status or tumor location distinguish one patient population from another. However, a greater understanding of these differences and similarities will allow researchers to more confidently draw conclusions about generalizability.

Another potential limitation of the current study was the sole focus on physical functioning-type impacts during the patient interviews. Although other types of impacts were spontaneously elicited and reported in the findings, these were not explored in as much depth, given time limitations and the focus of this study. The focus on physical functioning was driven from a PRO measurement perspective; previous literature indicated that it is a core contributor to patient HRQoL, and less susceptible to confounding sources other than the disease or its treatment [6], thus taking precedence over other impacts in terms of measuring drug efficacy.

Documenting and describing the relevant sign, symptoms, and impacts from the patient perspective contributes to a deeper understanding of the specific patient experience of HR+/HER2- advanced breast cancer and can provide avenues to identify and target the measurement of treatment outcomes. Recent emphasis on PROs from the FDA, particularly in oncology [4, 5], highlights the continued need to encourage and affirm the patient voice in drug development. Future research is necessary to both confirm the findings of this study and assess their applicability to the general advanced breast cancer population.

## Conclusions

Results of qualitative interviews with patients, supported by interviews with experienced oncologists, indicate that individuals with HR+/HER2- advanced breast cancer experience a myriad of concepts related to both disease and treatment, with fatigue/tiredness, hair loss, general pain, and lump in breast reported most frequently and shortness of breath, fatigue/tiredness, general pain, and vomiting reported as the most bothersome. Further, the results of the concept elicitation activities described herein indicate that HR+/HER2- advanced breast cancer affects individuals’ ability to function physically, such as walking, ability to do housework, and ability to cook, as well as impacting other areas of their lives (due to physical limitations or other attributes of their condition), such as family, social life, work, and emotional well-being.

## Additional files

Additional file 1: **Table S3.** HR+ and HER2- advanced breast cancer health and well-being impact concept descriptions. (DOCX 20 kb)

Additional file 2: **Table S1.** HR+ and HER2- advanced breast cancer disease-related and treatment-related concept frequencies and descriptions. (DOCX 17 kb)

Additional file 3: **Table S2.** HR+ and HER2- advanced breast cancer physical function impact concept frequencies and descriptions. (DOCX 17 kb)

## Abbreviations

FDA: Food and Drug Administration
HER2-: Human epidermal growth factor receptor-2 negative
HR+: Hormone receptor positive
HRQoL: Health-related quality of life
PRO: Patient-reported outcome

## References

[1] National Comprehensive Cancer Network (2017). Stage IV Breast Cancer Guidelines Version 1.0. https://www.nccn.org/patients/guidelines/stage_iv_breast/index.html#. Accessed 3/27/17.

[2] Smith I (2006). Goals of treatment for patients with metastatic breast Cancer. Seminars in Oncology.

[3] US Food and Drug Administration (2009). Guidance for industry on patient-reported outcome measures: Use in medical product development to support labeling claims; availability. Federal Register.

[4] Zagadailov E, Fine M, Shields A (2013). Patient-reported outcomes are changing the landscape in oncology care: Challenges and opportunities for payers. American Health & Drug Benefits.

[5] US Department of Health and Human Services, Food and Drug Administration, Center for Drug Evaluation and Research, & Center for Biologics Evaluation and Research (2015). Guidance for Industry: Clinical Trial Endpoints for the Approval of Non-Small Cell Lung Cancer Drugs and Biologics.

[6] Kluetz PG, Slagle A, Papadopoulos EJ, Johnson LL, Donoghue M, Kwitkowski VE (2016). Focusing on Core patient-reported outcomes in Cancer clinical trials: Symptomatic adverse events, physical function, and disease-related symptoms. Clinical Cancer Research.

[7] Garcia SF, Rosenbloom SK, Beaumont JL, Merkel D, Von Roenn JH, Rao D (2012). Priority symptoms in advanced breast cancer: Development and initial validation of the National Comprehensive Cancer Network-Functional Assessment of Cancer therapy-breast Cancer symptom index (NFBSI-16). Value Health.

[8] Lewis S, Yee J, Kilbreath S, Willis K (2015). A qualitative study of women's experiences of healthcare, treatment and support for metastatic breast cancer. Breast.

[9] Mosher CE, Johnson C, Dickler M, Norton L, Massie MJ, DuHamel K (2013). Living with metastatic breast cancer: A qualitative analysis of physical, psychological, and social sequelae. The Breast Journal.

[10] Turner J, Kelly B, Swanson C, Allison R, Wetzig N (2005). Psychosocial impact of newly diagnosed advanced breast cancer. Psychooncology.

[11] Krohe M, Hao Y, Lamoureux RE, Galipeau N, Globe D, Foley C (2016). Patient-reported outcomes in metastatic breast Cancer: A review of industry-sponsored clinical trials. Breast Cancer (Auckl).

[12] Turner-Bowker DM, Lamoureux RE, Stokes J, Litcher-Kelly L, Galipeau N, Yaworsky A (2018). Informing a priori sample size estimation in qualitative concept elicitation interview studies for clinical outcome assessment instrument development. Value Health.

[13] Patrick DL, Burke LB, Gwaltney CJ, Leidy NK, Martin ML, Molsen E (2011). Content validity-establishing and reporting the evidence in newly developed patient-reported outcomes (PRO) instruments for medical product evaluation: ISPOR PRO good research practices task force report: Part 1-eliciting concepts for a new PRO instrument. Value Health.

[14] Friese S (2013). ATLAS.ti 7 User Guide and Reference.

[15] Lewins A, Silver C (2007). Using software in qualitative research: A step-by-step guide.

[16] Lasch KE, Hassan M, Endicott J, Piault-Luis EC, Locklear J, Fitz-Randolph M (2012). Development and content validity of a patient reported outcomes measure to assess symptoms of major depressive disorder. BMC Psychiatry.

[17] Charmaz K, Smith JA, Harre R, Van Langenhove L (1995). Grounded theory. Rethinking methods in psychology.

[18] Glaser B, Strauss AL, Glaser B, Strauss AL (1967). The constant comparative method of qualitative analysis. Discovery of grounded theory: Strategies for qualitative research.

[19] Butt Z, Rosenbloom SK, Abernethy AP, Beaumont JL, Paul D, Hampton D (2008). Fatigue is the most important symptom for advanced cancer patients who have had chemotherapy. Journal of the National Comprehensive Cancer Network.

[20] Siefert ML (2010). Fatigue, pain, and functional status during outpatient chemotherapy. Oncology Nursing Forum.

[21] Cleeland CS, Sloan JA (2010). Assessing the Symptoms of Cancer Using Patient-Reported Outcomes (ASCPRO): searching for standards. Journal of Pain and Symptom Management.

[22] Rosenberg SM, Stanton AL, Petrie KJ, Partridge AH (2015). Symptoms and symptom attribution among women on endocrine therapy for breast Cancer. The Oncologist.

