# Supplementary material for: Understanding key symptoms, side effects, and impacts of HR+/HER2- advanced breast cancer: qualitative study findings
Source: J Patient Rep Outcomes. 2019 Feb 7;3:10. doi: 10.1186/s41687-019-0098-1 (PMC6367496; doi:10.1186/s41687-019-0098-1)
Supplement: Supplementary file 1 — Table S3. HR+ and HER2- advanced breast cancer health and well-being impact concept descriptions. (DOCX 20 kb) [file 41687_2019_98_MOESM1_ESM.docx]

| Table S3. HR+ and HER2- advanced breast cancer health and well-being impact concept descriptions | |
| --- | --- |
| **Concept** | Frequency of patient reports  N=15  n (%)^*^ |
| Emotional | |
| Depression | 1 (6.7%) |
| Fear | 1 (6.7%) |
| Frustration | 1 (6.7%) |
| Loss of identity | 1 (6.7%) |
| Nervousness | 1 (6.7%) |
| Social function/relationships | |
| Outings | 4 (26.7%) |
| Needs of family | 3 (20.0%) |
| Loss of intimacy with partner | 1 (6.7%) |
| Reduced desire to socialize | 1 (6.7%) |
| Work | |
| Reduced ability to work | 4 (26.7%) |

^*^Frequency is presented as the total count for each concept reported at least once by patients; all impacts were spontaneously reported by the patient without prior mention by the interviewer
