# Supplementary material for: Understanding key symptoms, side effects, and impacts of HR+/HER2- advanced breast cancer: qualitative study findings
Source: J Patient Rep Outcomes. 2019 Feb 7;3:10. doi: 10.1186/s41687-019-0098-1 (PMC6367496; doi:10.1186/s41687-019-0098-1)
Supplement: Supplementary file 2 — Table S1. HR+ and HER2- advanced breast cancer disease-related and treatment-related concept frequencies and descriptions. (DOCX 17 kb) [file 41687_2019_98_MOESM2_ESM.docx]

| Table S1. HR+ and HER2- advanced breast cancer disease-related and treatment-related concept frequencies and descriptions | |
| --- | --- |
| Concept | Frequency of patient reports^*^ N=15  n (%) |
| Fatigue/tiredness | 12 (80.0%) |
| Hair loss | 10 (66.7%) |
| General pain | 7 (46.7%) |
| Lump in breast | 7 (46.7%) |
| Nausea | 6 (40.0%) |
| Shortness of breath | 6 (40.0%) |
| Weakness | 5 (33.3%) |
| Diarrhea | 4 (26.7%) |
| Lymphedema | 4 (26.7%) |
| Neuropathy | 4 (26.7%) |
| Skin burn | 4 (26.7%) |
| Altered taste | 3 (20.0%) |
| Constipation | 3 (20.0%) |
| Feeling unwell | 3 (20.0%) |
| Injection site reaction | 3 (20.0%) |
| Joint pain | 3 (20.0%) |
| Loss of appetite | 3 (20.0%) |
| Vomiting | 3 (20.0%) |
| Weight loss | 3 (20.0%) |
| Bone pain | 2 (13.3%) |
| Dizziness | 2 (13.3%) |
| Headache | 2 (13.3%) |
| Hot flashes | 2 (13.3%) |
| Indent in breast | 2 (13.3%) |
| Memory loss | 2 (13.3%) |
| Mouth sores | 2 (13.3%) |
| Nail issues | 2 (13.3%) |
| Neck swelling | 2 (13.3%) |
| Stiffness | 2 (13.3%) |
| Stomach pain | 2 (13.3%) |
| Acid reflux | 1 (6.7%) |
| Allergic reaction | 1 (6.7%) |
| Bleeding | 1 (6.7%) |
| Bloating | 1 (6.7%) |
| Breast size decrease | 1 (6.7%) |
| Chemotherapy brain | 1 (6.7%) |
| Cough | 1 (6.7%) |
| Flu-like symptoms | 1 (6.7%) |
| Lung fluid build-up | 1 (6.7%) |
| Flushing | 1 (6.7%) |
| Gout | 1 (6.7%) |
| Itching | 1 (6.7%) |
| Lack of balance | 1 (6.7%) |
| Lymph node inflammation | 1 (6.7%) |
| Runny nose | 1 (6.7%) |
| Skin peeling | 1 (6.7%) |
| Sore throat | 1 (6.7%) |
| Vaginal bleeding | 1 (6.7%) |
| Weight gain | 1 (6.7%) |

^*^Frequency is presented as the total count for each concept reported at least once by patients; all signs, symptoms, and side-effects were spontaneously reported by the patient without prior mention by the interviewer
