# Supplementary material for: Understanding key symptoms, side effects, and impacts of HR+/HER2- advanced breast cancer: qualitative study findings
Source: J Patient Rep Outcomes. 2019 Feb 7;3:10. doi: 10.1186/s41687-019-0098-1 (PMC6367496; doi:10.1186/s41687-019-0098-1)
Supplement: Supplementary file 3 — Table S2. HR+ and HER2- advanced breast cancer physical function impact concept frequencies and descriptions. (DOCX 17 kb) [file 41687_2019_98_MOESM3_ESM.docx]

| Table S2. HR+ and HER2- advanced breast cancer physical function impact concept frequencies and descriptions | |
| --- | --- |
| Concept | Frequency of patient reports  N=15  n (%)^*^ |
| **Physical function impacts** | |
| Activities of daily living (ADLs) | |
| Toileting | 2 (13.3%) |
| Bathing | 1 (6.7%) |
| Instrumental ADLs | |
| Ability to do housework | 11 (73.3%) |
| Ability to cook | 8 (53.3%) |
| Ability to shop | 6 (40.0%) |
| Ability to drive | 4 (26.7%) |
| General physical functioning | |
| Ability to walk | 11 (73.3%) |
| Ability to lift | 4 (26.7%) |
| Ability to climb stairs | 3 (20.0%) |
| Ability to exercise | 3 (20.0%) |
| Ability to lie down | 3 (20.0%) |
| Ability to sit | 3 (20.0%) |
| Ability to stand | 3 (20.0%) |
| Fine motor skills | 2 (13.3%) |
| Ability to bend | 1 (6.7%) |
| Ability to get in and out of car | 1 (6.7%) |
| Ability to move at normal pace | 1 (6.7%) |
| Ability to stand from seated position | 1 (6.7%) |
| Leisure activities | |
| Dancing | 3 (20.0%) |
| Gardening | 3 (20.0%) |
| Bowling | 2 (13.3%) |
| Crocheting/  Knitting | 1 (6.7%) |
| Ability to care for pets | 1 (6.7%) |
| Ability to play pool | 1 (6.7%) |
| Lack of interest in hobbies | 1 (6.7%) |
| Sleep | |
| Ability to sleep | 4 (26.7%) |

^*^Frequency is presented as the total count for each concept reported at least once by patients; all impacts were spontaneously reported by the patient without prior mention by the interviewer
